# Insecticidal, Repellent and Antifeedant Activity of Essential Oils from *Blepharocalyx cruckshanksii* (Hook. & Arn.) Nied. Leaves and *Pilgerodendron uviferum* (D. Don) Florin Heartwood against Horn Flies, *Haematobia irritans* (Diptera: Muscidae)

**DOI:** 10.3390/molecules26226936

**Published:** 2021-11-17

**Authors:** Javier Espinoza, Cristian Medina, Washington Aniñir, Paul Escobar-Bahamondes, Emilio Ungerfeld, Alejandro Urzúa, Andrés Quiroz

**Affiliations:** 1 Laboratorio de Ecología Química, Departamento de Ciencias Químicas y Recursos Naturales, Universidad de La Frontera, Casilla 54-D, Avenida Francisco Salazar 01145, Temuco 4811230, Chile; cristian.medina@ufrontera.cl (C.M.); w.aninir01@ufromail.cl (W.A.); 2 Centro de Excelencia en Investigación Biotecnológica Aplicada al Medio Ambiente (CIBAMA), Universidad de La Frontera, Avenida Francisco Salazar 01145, Temuco 4811230, Chile; 3 Doctorado en Ciencias de Recursos Naturales, Universidad de La Frontera, Av. Francisco Salazar 01145, Temuco 4811230, Chile; aurzuamoll44@yahoo.com; 4 Centro Regional de Investigación Carillanca, Vilcún, Instituto de Investigaciones Agropecuarias (INIA), Región de La Araucanía, Temuco 7500502, Chile; paul.escobarc@inia.cl (P.E.-B.); emilio.ungerfeld@inia.cl (E.U.)

**Keywords:** horn flies, *Haematobia irritans*, *Blepharocalyx cruckshanksii*, *Pilgerodendron uviferum*, essential oils, repellency, mortality, antifeedancy

## Abstract

*Haematobia irritans* is a cosmopolitan obligate blood-feeding ectoparasite of cattle and is the major global pest of livestock production. Currently, *H. irritans* management is largely dependent on broad-spectrum pesticides, which has led to the development of insecticide resistance. Thus, alternative control methods are needed. Essential oils have been studied as an alternative due to their wide spectrum of biological activities against insects. Thus, the main aim of this study was to evaluate the insecticidal, repellent and antifeedant activity of the essential oils from *Blepharocalyx cruckshanksii* leaves and *Pilgerodendron uviferum* heartwood against horn flies in laboratory conditions. The composition of the essential oils was analyzed using gas chromatography coupled to mass spectrometry. Accordingly, α-pinene (36.50%) and limonene (20.50%) were the principal components of the *B. cruckchanksii* essential oil, and δ-cadinol (24.16%), cubenol (22.64%), 15-copaenol (15.46%) and δ-cadinene (10.81%) were the most abundant compounds in the *P. uviferum* essential oil. Mortality of flies and feeding behavior were evaluated by non-choice tests, and olfactory response was evaluated using a Y-tube olfactometer. Both essential oils were toxic to horn flies, with LC_50_ values for *B. cruckchanksii* essential oil of 3.58 µL L^−1^ air at 4 h, and for *P. uviferum* essential oil of 9.41 µL L^−1^ air and 1.02 µL L^−1^ air at 1 and 4 h, respectively. Moreover, the essential oils exhibited spatial repellency in the olfactometer using only 10 µg of each oil, and these significantly reduced the horn fly feeding at all doses evaluated. Although further laboratory and field studies related to the insectistatic and insecticide properties of these essential oils against *H. irritans* are necessary, *B. cruckshanksii* leaves and *P. uviferum* heartwood essential oils are promising candidates for horn fly management.

## 1. Introduction

*Haematobia irritans irritans* (L.) (Diptera: Muscidae), commonly known as the horn fly, is a cosmopolitan obligate blood-feeding ectoparasite of cattle grazing [1,2], and it is considered one of the major global pests in livestock production [3]. It is a little brown-gray fly between 2 and 5 mm in length, which makes it the smallest biting fly that attacks beef cattle [4,5]. *H. irritans* is widely distributed in tropical, subtropical, and some temperate regions of the Northern Hemisphere [6], including Europe, Asia Minor, and North Africa. In the Americas, *H. irritans* ranges from southern Canada to temperate areas of Argentina, Uruguay, and Chile [7,8,9,10]. The first colonies of horn flies in Chile were found in the late sixties, but the species was definitively established in 1993 [10,11]. In Chile, *H. irritans* is found throughout the country between the Regions of Arica y Parinacota and Aysén and the adult fly emerges from November to May, showing a population peak in summer (December to March) [11]. However, because of climate change, seasonality has decreased, and the summer season has been extended [12]. Both, male and female *H. irritans* use their piercing proboscis to feed on cattle, typically 24–38 times per day [1,13,14]. The fly feeding activity causes extreme annoyance to cattle, which spend energy in defensive behaviors, principally, head and tail toss, bunching and seeking refuge to keep the flies off their bodies resulting in elevated heart and respiratory rates, and reduced grazing time, especially when the animals suffer a massive infestation (>200 horn flies per animal) [15,16,17]. This provokes a reduction in sleeping time and worsening of feed conversion efficiency, reduced milk production and weight gain [13,17,18,19,20]. One beef cow can be parasitized by up to 4000 horn flies [6]. It has been estimated that for every 100 flies per animal, the calf weight gain is reduced by 8.1 kg per animal per year [21]. With over 500 flies per animal, one beef cattle can lose approximately 0.25 kg daily [6], whereas dairy cow milk yield can decline by 20% [22,23]. Another important aspect of fly biting concerns skin damage, including fiber separation and scarring, which leads to reduced leather quality and lower commercial value [21]. Moreover, horn flies are a vector of *Staphylococcus aureus*, a causative agent for mastitis, bovine teat atresia [24,25,26], and they are the intermediate host for *Stephanofilaria stilesi*, a filarial nematode that causes skin lesions on cattle [13].

In the United States, the damages caused by *H. irritans* cost USD 1 billion each year, including costs of chemical management [1,13,14,27], and in Brazil, annual losses were estimated to be from USD 150 million to USD 2.56 billion in 2012 [28]. In Chile, the annual economic losses caused by the horn fly were evaluated at CLP 25,800 million [29,30], equivalent to USD 45 million in January 2001 [31]. This estimation did not consider losses produced by reduced reproductive efficiency and leather quality [32].

Management of *H. irritans* is largely dependent upon broad-spectrum pesticides, mainly organophosphates and pyrethroids. Unfortunately, pesticides are highly persistent, harmful to the environment; they have significant residual toxicity and have led to the development of insecticide resistance [20,33,34]. Thus, to avoid these problems, environmentally friendly methods of control are necessary [31]. The use of horn fly-resistant cattle breeds [19,35,36], horn fly natural predators [21,37,38], coprophagous beetles [39,40,41], entomopathogenic fungi [40,41,42,43,44,45,46], grasses infected with endophytic fungi [47,48] and their isolated active compounds [31,49,50], and novel vaccines [21] have been investigated as control alternatives. However, field implementation of those alternative control methods has so far been limited. Consequently, the search for products for controlling insect pests, such as plant-derived natural products appears as an attractive ecological and natural alternative to be explored. Plants produce chemical compounds involved in defense mechanisms against the attack of microorganisms and predators. Among those plant-natural products, essential oils (EOs) have been used for hundreds of years to protect humans and animals from arthropod attack [51]. Essential oils are complex mixtures of volatile secondary metabolites, mostly monoterpenes and sesquiterpenes in addition to other minor constituents, which are biosynthesized in aromatic plants [52]. EOs have a wide spectrum of biological activities against bacteria, fungi, viruses, and insects [53,54]. Certainly, the study and application of the EOs is an interesting alternative to develop safe and eco-sustainable insecticides and repellents of *H. irritans* [54]. Although the activity of essential oils against insects has been well documented [52,54], relatively little have been tested against horn flies [55,56,57,58,59,60,61]. *H. irritans* has been reported to be susceptible mainly to well-known EOs obtained from *Carapa guianensis* Aubl. (Meliaceae) [57], *Mentha × piperita* L. (Lamiaceae) [58], *Ocimum basilicum* L. (Lamiaceae) [58], *Lavandula angustifolia* Mill. (Lamiaceae) [58], *Cymbopogon citratus* (D.C.) Stapf. (Poaceae) [58], *Pelargonium* spp. (Geraniaceae) [58], *Pinus sylvestris* L. (Pinaceae) [58], *Schinus molle* Rev L. (Anacardiaceae) [59], *Cinnamomum verum* J. Presl (*C. zeylanicum*) (Lauraceae) [60], *Eucalyptus* spp. (Myrtaceae) [61], and *Melaleuca alternifolia* (Maiden. and Betche.) Cheel. (Myrtaceae) [57,62]; though there is no information about the effect of endemic *Blepharocalyx cruckshanksii* (Hook. & Arn.) Niedenzu and *Pilgerodendron uviferum* (D. Don) Florin essential oils on the horn flies. The flora of southern Chile is characterized by a high degree of endemism and is a potential resource for obtaining EOs with insecticidal and insectistatic effects against the horn fly.

*Pilgerodendron uviferum* (D. Don) Florin (Cupressaceae), commonly named as Ciprés de las Guaitecas or Lahuán [63], is a conifer native to Southern Chile and Argentina, and is the only representative of the Pilgerodendron genus [64]. It is characteristic of wet areas and poorly drained sites with high annual rainfall. It is a slow-growing, narrowly conical evergreen tree that can reach trunk diameters of up to 1.1 m, heights of up to 40 m and ages of more than 500 years. The leaves are scale-like and arranged in decussate pairs [63,64]. *P. uviferum* is known to be highly resistant to attack by microorganisms and insects. It has been postulated that its resistance is related to its chemical composition [65,66]. In fact, recent studies have shown a repellent effect for the *P. uviferum* heartwood EO against the weevil *Aegorhinus superciliosus*, a major berry pest in Chile [67]; antifeedant effects of the EO, extracts and isolated compounds from heartwood against *Hylastinus obscurus* [68], one of the most important pests of red clover [69,70]; and antibacterial properties against antibiotic resistant *S. aureus* [71]. 

*Blepharocalyx cruckshanksii* (Hook. & Arn.) Niedenzu (Myrtaceae), commonly named Temo [72,73], is an infrequent species of perennial leaves, endemic to Chile that inhabits in moist forests between 34° and 42° South Latitude. The plant grows to 15 m high with a trunk diameter of approximately 50 cm. Its bark is smooth and reddish brown. The leaves are oval-shaped, while the flowers are white and arranged in inflorescences. The fruits are round, dark brown with hints of reddish tone, and bitter taste [72,74]. Different tissues of *B. cruckshanksii* have been used as astringents, to treat wounds, diarrhea, and rheumatism [73,75,76]. Moreover, compounds isolated from bioactive fractions of *B. cruckshanksii* were observed to have antimycobacterial activity [76]. Additionally, in a recent study, an extract of *B. cruckshanksii* bark showed cytotoxic activity [77]. Despite the above, *B. cruckshanksii* biological activities have not been well-studied and no investigation has been carried out to explore the anti-insect properties of this species. *Blepharocalix salicifolius* (Kunth) O. Berg, endemic to southern Brazil, Argentina, Paraguay, and Uruguay has been studied end extracts of this species have shown antiparasitic, antifungal, antibacterial, allelopathic, cytotoxic and insecticide effects [77]. 

Considering the above, *P. uviferum* and *B. cruckshanksii* may be potential sources of natural products for the horn fly control. Therefore, the main aim of this study was to evaluate the insecticidal, repellent and antifeedant activity of the essential oils from *B. cruckshanksii* leaves and *P. uviferum* heartwood against *H. irritans* adults in laboratory conditions.

## 2. Results

Fresh *B. cruckchanksii* leaves (0.96 kg) were milled and hydrodistillated to yield 1.27 g (1.31 mL) of a pale-yellow EO (0.13% *w*/*w*). The EO was analyzed by GC/MS (Table 1). Twenty-nine compounds were identified in the EO, corresponding to all detected compounds. Four non-oxygenated monoterpenes (58.18%) and 25 sesquiterpenes (41.82%)—19 non-oxygenated (37.52%) and six oxygenated (4.30%)—were present in the EO. The monoterpene hydrocarbons α-pinene (36.50%) and limonene (20.50%) were the most abundant compounds in the EO from *B. cruckchanksii* leaves, followed by the sesquiterpene hydrocarbons, calamenene (8.69%), cubenene (5.65%) and caryophyllene (4.63%) (Table 1). Identification was corroborated by comparison of the obtained mass spectra with those of the literature, retention indexes and co-injection with standards. The EO composition of *P. uviferum* heartwood was obtained from Espinoza et al. [67]. According to these analyses, three monoterpenes (0.43%) and 17 sesquiterpenes (86.62%) were present in EO, with torreyol (24.16%), cubenol (22.64%), 15-copaenol (15.46%), and δ-cadinene (10.81%) being the most abundant components [67].

In the toxicity bioassay, both essential oils demonstrated toxic effects against horn flies. The *B. cruckchanksii* EO at 7.73 µL L^−1^ air (0.86 mg of EO) and 3.87 µL L^−1^ air (0.43 mg of EO) were the lowest concentrations, significantly different (*p* ≤ 0.05) from the blank, able to kill of flies at 1 h (11.11 ± 1.26 × 10^−15^% of dead flies) and 4 h (59.26 ± 3.70% of dead flies) after application, respectively. The *B. cruckchanksii* EO at 15.46 µL L^−1^ air (1.72 mg of EO) was able to kill the greatest number of flies at 1 h (13.33 ± 3.33% of dead flies) and 4 h (80.00 ± 3.33% of dead flies) post treatment. The median lethal concentration (LC_50_) value calculated by *B. cruckchanksii* EO using a Probit analysis was 3.58 µL L^−1^ air at 4 h (Table 2). The LC_50_ value at 1 h was not determined, since the mortality registered for the highest tested concentration was no more than 13.33%. The *P. uviferum* EO at 1.85 µL L^−1^ air (0.175 mg of EO) and 0.37 µL L^−1^ air (0.035 mg of EO) were the lowest concentrations, significantly different (*p* ≤ 0.05) from the blank, capable to kill of flies after 1 h (18.15 ± 4.07% of dead flies) and 4 h (30.20 ± 1.75% of dead flies) of exposure, respectively. The *P. uviferum* EO at 11.1 µL L^−1^ air (1.05 mg of EO) was able to kill the greatest number of flies at 1 h (56.67 ± 3.33% of dead flies) and 4 h (93.33 ± 3.33% of dead flies). The LC_50_ values for *P. uviferum* EO were 9.41 µL L^−1^ air at 1 h. With the increased exposure time to 4 h, the LC_50_ values decreased to 1.02 µL L^−1^ air (Table 2). 

**Figure 1:**
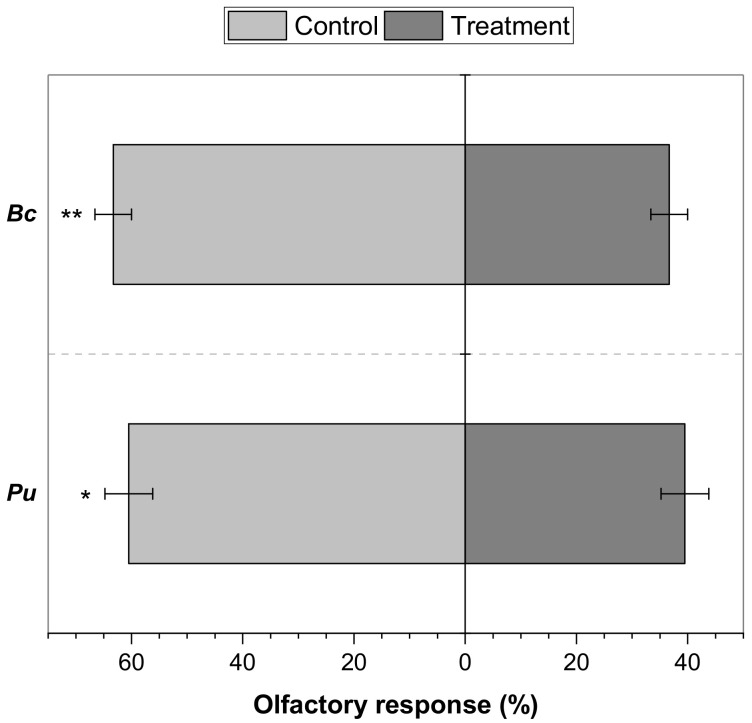
Olfactory response of horn flies to *B. cruckchanksii* leaves and *P. uviferum* heartwood EOs. Values indicate the mean + SE. Asterisks on the bars indicate significant differences between the treatments and control based on the Tukey HSD test (* *p* ≤ 0.05, ** *p* ≤ 0.01), *N* = 60.

In the olfactory test, the Y-tube olfactometer allowed to prove the repellency provoked by the two essential oils tested (Figure 1). *B. cruckchanksii* leaves EO and *P. uviferum* heartwood EO exhibited spatial repellency in the olfactometer, where 63.3% and 60.5% of the individuals chose the control, respectively. It was different and highly significant from the 36.7% of flies that chose the *B. cruckchanksii* EO (*p* ≤ 0.01), and significantly different from the 39.5% of flies that chose *P. uviferum* EO (*p* ≤ 0.05).

**Figure 2:**
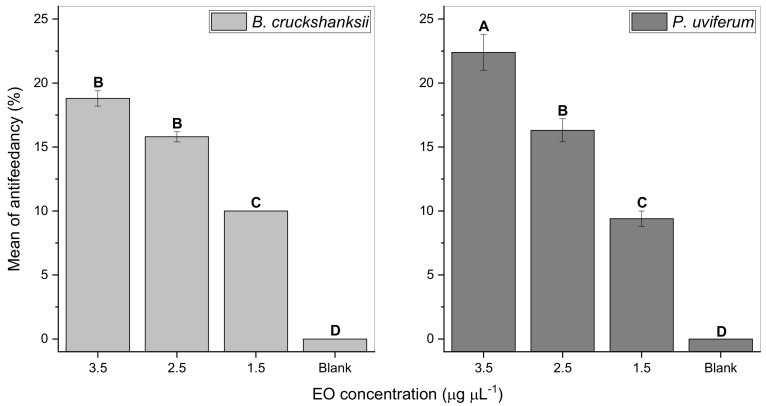
Antifeedancy levels of the *B. cruckchanksii* leaves and *P. uviferum* heartwood EOs against horn flies at three concentrations (1.5, 2.5, and 3.5 µg µL^−1^) in laboratory bioassays compared to a blank. Values indicate the mean + SE. Letters on the columns indicate significant differences between the treatments and the blank based on the Tukey HSD test (*p* ≤ 0.05), *N* = 30.

In the antifeedant assay (Figure 2), both EOs showed antifeedant activities and a concentration-dependent response was observed. Fly mortality was recorded during the bioassays, but significant differences were not observed in relation to the control. Data analysis showed that the interaction among all the variables was significant (F = 3.66; df = 3, 16; *p* < 0.05). Significant differences were observed among the three treatment doses and the blank (F = 333.66; df = 3, 16; *p* < 0.05). However, the interaction among both EOs was not significant (F = 3.33; df = 1, 16; *p* > 0.05). The *B. cruckchanksii* leaves and *P. uviferum* heartwood EOs significantly reduced horn fly feeding at all doses evaluated. Particularly, *P. uviferum* EO showed 22.4%, 16.3%, and 9.4% antifeedancy at 3.5, 2.5, and 1.5 μg μL^−1^, respectively. Antifeedancy was significantly different among each dose. *B. cruckchanksii* EO generated 18.8%, 15.8%, and 10.0% antifeedancy at 3.5, 2.5, and 1.5 μg μL^−1^, respectively. Antifeedancy at 3.5 and 2.5 μg μL^−1^ did not differ significantly, but it was significantly different from antifeedancy at 1.5 μg μL^−1^. All treatment was significantly different from the blank.

Comparing among treatments, *P. uviferum* EO at 3.5 µg µL^−1^ concentration produced the largest antifeedant activity. *B. cruckchanksii* EO at 3.5 and 2.5 μg μL^−1^, and *P. uviferum* EO at 2.5 µg µL^−1^ were not different among each other and were more active than *B. cruckchanksii* and *P. uviferum* EOs at 1.5 µg µL^−1^; this being statistically equal among each other but significantly different from blanks.

## 3. Discussion

In the small genus *Blepharocalyx*, consisting of only three species [78]; *B. cruckshanksii*, *B. eggersii*, and *B. salicifolius*; all studied EO samples are rich in monoterpenes [78], and the EO analyzed here is not the exception. The monoterpenes α-pinene and limonene were the most abundant compounds in the EO of *B. cruckchanksii* leaves, which is in accordance with the only report about the composition of the *B. cruckchanksii* EO [79]. In this, two EOs of leaves collected from different populations of *B. cruckchanksii* in Chile were principally constituted by limonene (51.65% and 13.86%) and α-pinene (17.28% and 15.60%), with lesser amounts of spathulenol (7.57% and 11.42%) [79]. Although the EO analyzed in the present study produced a higher amount of α-pinene (36.50%) than limonene (20.50%), and spathulenol was a minor component (0.81%) (Table 1). Particularly, the *B. cruckchanksii* EO analyzed in the present study was more similar to the composition of one of the two EOs previously studied. Both EOs showed a monoterpene/sesquiterpene relation of around 1:1; instead, the other EO has a monoterpene/sesquiterpene relation of 8:2. Regrettably, the authors did not report more information about retention indexes, sampling conditions and location. Thus, a further comparison was not possible. The EO of *P. uviferum*, which is the only representative of the *Pilgerodendron* genus, were almost exclusively composed of sesquiterpenes (99.5%) [67], in accordance with the report of Oyarzún and Garbarino [80]. However, the composition was very different from the *P. uviferum* EO from leaves [81], which was rich in monoterpenes (54.1%), but with a large presence of sesquiterpenes (40.4%) [67]. 

The insecticidal, repellent and antifeedant activity of EOs from *B. cruckshanksii* leaves and *P. uviferum* heartwood against horn flies were evaluated here, in laboratory conditions. Both EOs showed strong insecticidal properties (Table 2) and exhibited a significant repellency (Figure 1) and antifeedancy (Figure 2) on the horn fly. Nevertheless, both EOs provoked poor antifeedant activities. It is the first report about the effects of *B. cruckshanksii* and *P. uviferum* EOs against *H. irritans*. 

The horn fly has shown susceptibility to EOs from other species, including some Myrtaceae plants [57,58,59,60,61,62]. *Carapa guianensis* Aubl. (Meliaceae) and *Melaleuca alternifolia* (Maiden. and Betche.) Cheel. (Myrtaceae) EOs, constituted mainly of α-humulene, bicyclogermacrene and germacrene-D (53.34%), and terpinen-4-ol and γ-terpinene (62.13%), respectively, showed insecticidal activity on horn flies in an in vitro study [57]. Both hand-sprayer EOs was able to kill 100% of flies using 100 µL of EO solutions at 1.0% (0.5 mg of EO per liter of air) and 5.0% (2.5 mg of EO per liter of air) for up to 4 h of exposure [57]. However, the LC_50_ values were not determined in this study. The repellent effect of the *C. guianensis* and *M. alternifolia* EOs at concentrations of 5.0% (0.5 g of EO in 10 mL of solution) was evaluated in vivo on Holstein cows infested by *H. irritans*. Both hand-sprayer EOs showed repellency at 1, 2 and 24 h, when the number of flies on cows treated with EOs was significantly lower than the number of flies on control animals [57]. The repellent effect of the same EOs mentioned above at concentrations of 5.0% (0.1 g of EO in 2 mL of solution) against horn flies was also evaluated in vitro. The EOs showed significant repellent activity [62]. It should be noted that the EOs compositions are quite different from the composition of the *B. cruckshanksii* and *P. uviferum* EOs. Moreover, in the present study, the biological activities depend on the volatility of each EO in the flasks during the assay, contrary to the abovementioned studies.

The EOs obtained from 16 species of *Eucalyptus* (Myrtaceae) by hydrodistillation were also evaluated against *H. irritans* [61]. These were mainly composed of 1,8-cineole, α-pinene, α-terpineol, 4-terpineol, and *p*-cymene. Vapors from these EOs and their major components were found to be toxic to *H. irritans* adults. The essential oil of *Eucalyptus polybractea* R. Baker, which contained 85.01% of 1,8-cineole, had the highest knockdown activity of 3.44 min to kill the half population of flies (KT_50_). Pure 1,8-cineole, *p*-cymene, and α-pinene at 46.32 µL L^−1^ air showed KT_50_ values of 3.92, 6.16, and 24.89 min, respectively. A correlation between the content of 1,8-cineole and *p*-cymene in the *Eucalyptus* EOs and the toxic effect was observed separately. However, no correlation between the α-pinene content and KT_50_ values was observed [61]. In the present study, 1,8-cineole and *p*-cymene were absent from the *B. cruckshanksii* EO and these were minor constituents in *P. uviferum* EO (1,8-cineole: 0.06%, and *p*-cymene: 0.12% [67]), even so, those EOs showed strong insecticidal properties. The *B. cruckshanksii* EO, which was mainly constituted by α-pinene (36.5%), was able to kill only 13.3% flies at 1 h using 15.46 µL L^−1^ air, corresponding to 5.64 µL of α-pinene per liter of air, a dose 8.2 times lower than pure α-pinene, although this was evaluated at different time. Therefore, the synergistic or antagonistic effect among the EOs constituents should be considered.

Essential oils from plants, which do not belong to the Myrtaceae family, have also been tested against horn flies [58,59,60,62]. *Schinus molle* Rev L. (Anacardiaceae) leaves essential oil, principally consisting of sabinene (48.63–51.74%), limonene (10.20–16.98%) and bicyclogermacrene (18.12%), was able to kill 59.4% of horn flies at 2 h post-application at 1 mg/mL in acetone. Using a different methodology, the same EO at 30 mg/mL in acetone (86.6 mg L^−1^ air) was able to kill 96% and 100% of flies at 2 h and 4 h, respectively. Microcapsule formulations of *S. molle* EO were also tested. The formulations showed lower activity due to the slow liberation of the EO (32% and 73% of dead flies at 2 h and 4 h, respectively) [59]. However, the LC_50_ values were not determined. In the present study, *B. cruckshanksii* EO and *P. uviferum* EO produced 80.0% and 93.3% of dead flies at 4 h, using a dose 6.5 and 10.7 times lower, respectively.

Repellent activity of seven plant essential oils against horn flies were tested in field conditions [58]. Basil (*Ocimum basilicum* L. (Lamiaceae)), geranium (*Pelargonium* spp. (Geraniaceae)), lavender (*Lavandula angustifolia* Mill. (Lamiaceae)), peppermint (*Mentha × piperita* L. (Lamiaceae)), lemongrass (*Cymbopogon citratus* (D.C.) Stapf. (Poaceae)) and pine (*Pinus sylvestris* L. (Pinaceae)) essential oils mixed with either sunflower oil or ethyl alcohol, were applied at 5% concentrations to the sides of pastured cows and barn-held heifers. Only basil, geranium, lavender, lemongrass, and peppermint EOs–sunflower oil repelled more flies than the carrier oil alone after 1.5 to 4 h of application on barn-held heifers. Lemongrass and pine EOs mixed with ethyl alcohol showed repellency > 62.9% during 0.5 to 6 h after treatment on pastured cows, but EOs–Ethanol mixtures were less repellent than EOs–carrier oil mixtures [58]. In a similar field study, 50 mL of *Cinnamomum* *verum* J. Presl *(C. zeylanicum*) (Lauraceae) EO diluted at 5% in surfactant solution non-ionic Triton^®^, significantly reduced the horn fly number on cows after 1, 2, 3, 9, 24 and 33 h of treatment [60]. Regrettably, the analyses of the EOs composition and their relationship with the repellent effects were not discussed in these studies.

On the antifeedant effect against horn flies, only four studies have been published [31,51,82,83]. Showler and Harlien [82] reported that *p*-anisaldehyde completely deterred feeding on cotton pads soaked in bovine blood using 0.5 mL of *p*-anisaldehyde solutions at 0.6 to 10% in acetone, but horn flies were not repelled by *p*-anisaldehyde in the olfactometers. Laboratory-grade limonene and a commercial limonene-based insecticide did not show antifeedancy in the same bioassay [83], but those products were attractants to horn flies at concentrations < 0.1%. In the present study, limonene was the second most abundant compound in the EO from *B. cruckchanksii* leaves (20.50%), which produced low antifeedancy (18.8%) at the highest dose tested—almost 9 to 143 times lower than doses used by Showler and Harlien [82]. Contrary to the reported [83], *B. cruckchanksii* EO exhibited a very significant repellency in the olfactometer, using 10 μL of EO at 1 μg μL^−1^ in acetone. This indicates that the effect of a particular terpene cannot be compared with the effect of a complex mixture of compounds as in the case of EOs. In another study, Zhu et al. [51] reported that catnip oil containing 85% (*Z*,*E*)- and (*E*,*Z*)-nepetalactone, geraniol constituted by >90% (2*E*)-3,7-dimethylocta-2,6-dien-1-ol, and a 1:1:1 mixture of octanoic, nonanoic, and decanoic acids (C8910 acids) reduced horn fly feeding and exhibited repellency in laboratory conditions, where at least 85% of the flies did not feed when exposed to 0.2 or 2 mg of repellent. Recently, the antifeedant and repellent activity of loline alkaloids against horn flies were tested in laboratory conditions. All samples showed antifeedant activities above 35.5% using al least 0.05 mg of sample [31]. In the present study, *B. cruckshanksii* and *P. uviferum* EOs reduced horn fly feeding. However, only 18.8% and 22.4% of the flies did not feed when exposed to 0.35 mg of *B. cruckshanksii* EO and *P. uviferum* EO, respectively (Figure 2). Although the antifeedancy of both EOs was low when it is compared with the abovementioned studies, this is the first report of the antifeedant effects of the *B. cruckshanksii* and *P. uviferum* EOs against *H. irritans*. Moreover, this is the first information on the antifeedancy of natural and non-commercial essential oils against the horn fly.

In summary, in the present study, the essential oil obtained from *B. cruckshanksii* leaves and *P. uviferum* heartwood were toxic, repellent and antifeedant agents against horn flies. These kinds of natural products, safe and eco-friendly are suitable candidates for horn fly management, although further laboratory and field studies related to the insectistatic and insecticide properties of the essential oils against *H. irritans* are necessary.

## 4. Materials and Methods 

### 4.1. H. Irritans Collection

Laboratory bioassays were conducted using wild horn flies collected from beef cattle according to Espinoza et al. [31]. Teen steers fed on endophyte-free tall fescue pastures were placed in a corridor at Centro Regional de Investigación INIA Carillanca, Vilcún, Chile (38°41′ SL, 72°25′ WL, 200 masl). Then, horn flies on steers were hand-collected and caught in a 1 L glass flask per bovine (50–60 flies per flask). Flasks were subsequently sealed with an entomological net and were transported to the laboratory. Once in the laboratory, the horn flies were pooled together and maintained at 25 ± 2 °C with variable humidity (30–50%), under a light:dark photoperiod of 12:12 h. 

### 4.2. Plant Material and Essential Oil Extraction

Fresh *B. cruckchanksii* leaves were collected in January 2020 in the south of the Quepe River Basin (38°50′28.7″ SL, 78°38′00.3″ WL, 200 masl), La Araucanía Region, Freire, Chile. The samples were identified by comparing macroscopic and microscopic morphologic characteristics to Chilean flora and specimens. A specimen was kept in the Laboratorio de Ecología Química de la Universidad de La Frontera, Chile. Leaves were cleaned with distilled water to remove any residue. The EO was extracted from 0.96 kg of fresh-milled *B. cruckchanksii* leaves by hydrodistillation for 4 h in a Clevenger-type apparatus and the resulting EO was dried over anhydrous sodium sulfate. The *P. uviferum* EO that was used here was produced in an earlier study by Espinoza et al. [67].

### 4.3. Essential Oil Analysis by GC/MS

Analysis of the *B. cruckchanksii* EO components was performed by gas chromatography/mass spectrometry (GC/MS) using the following instrumentation: a Thermo Electron Model Focus GC (Waltham, MA, USA) coupled to a DSQ Thermo Electron quadrupole mass spectrometric detector, with an integrated data system (Xcalibur 2.0, Thermo Fisher Scientific Inc., Waltham, MA, USA) and a 30 m length BPX5 capillary column (0.25 μm film thickness × 0.25 mm i.d., SGE Forte, Trajan Scientific and Medical, Ringwood, VIC, Australia). The operating conditions were as follows: on-column injection; injector, transfer line, and detector temperature, 250 °C; oven temperature program: Hold at 40 °C for 1 min. Subsequently, it was increased to 250 °C at 5 °C min^−1^, and then maintained for 5 min. He at 1.00 mL min^−1^ was used as carrier gas. The mass spectra were obtained at an ionization voltage of 70 eV. The recording conditions employed a scan time of 1.5 s and a mass range of 30 to 400 amu. The identification of compounds in the chromatographic profiles was achieved by comparison of their mass spectra with those in the NIST ver. 2.0 library database (NIST, Gaithersburg, MD, USA), in some cases, by comparison of their retention times and mass spectra with those of standards [68], and by comparison of their calculated retention index with those reported in the literature [84] for the same type of stationary phase. Calculated retention indices were determined by means of the retention time of C9–26 *n*-alkane standards (100 µg mL^−1^ in hexane) (Sigma-Aldrich, St. Louis, MO, USA) using the equation described by Kovats and Keulemans [85]. Analyses of the *P. uviferum* EO compounds had previously been performed using gas chromatography (GC) and gas chromatography/mass spectrometry (GC/MS) [67].

### 4.4. Laboratory Feeding Bioassay

#### 4.4.1. Blood Collection

The blood samples were taken from 15 steers (4.5 mL per animal) fed on endophyte-free tall fescue pastures by puncture of the jugular vein using 13 × 75 mm, 4.5 mL BD Vacutainer*^®^* glass tubes (Becton Dickinson, Franklin Lakes, NJ, USA) containing buffered sodium citrate solution (3.2%, 0.109 M). The blood samples were transferred to the laboratory in coolers at a temperature close to 4–7 °C. Once in the laboratory, the blood samples were pooled together in a sterile glass flask and kept at 4 °C in a refrigerator [31].

#### 4.4.2. Feeding Bioassay

The antifeedancy of the EOs was tested by a no-choice test according to Zhu et al. [51] with modifications [31]. Horn flies collected according to Section 4.1 were starved for 24 h prior to testing. Doses (100 μL) of EOs dissolved in acetone (liquid chromatography grade; LiChrosolv^®^, Merck KGaA, Darmstadt, Germany) at 1.5, 2.5, and 3.5 µg µL^−1^ concentrations were applied on individual tulle pieces (4 × 5 cm) to each EO and concentration. After the solvent evaporated (2–3 min), each sample-impregnated layer was separately placed on top of a blood-soaked cotton pad, and it was put into a glass flask (4 × 5 × 6.45 cm). A control sample was treated with 100 μL of acetone only. Horn flies were transferred into each testing flask (10 flies per flask). After 4 h, the mortality was recorded, and the flies were frozen at −20 °C for 1 h. Flies were then checked for feeding status by squashing their abdomens and examining under microscope (20×) for the presence of blood. The experiments were performed in triplicate for each concentration. The antifeeding performance was evaluated according to Equation (1):

(1) $$Mean\;of\;antifeedancy(\%)=\frac{\left(Unfed\;flies\times 100\right)}{Total\;flies}$$

#### 4.4.3. Toxicity Bioassays

The toxicity of the EOs was tested by a no-choice test according to Tampe et al. [86] with modifications [31]. Briefly, different doses of EOs were applied on individual tulle pieces (4 × 5 cm). Each sample-impregnated layer was separately placed on the bottom of a glass jar (4 × 5 × 6.45 cm) to obtain a final concentration of 11.1, 7.4, 5.6, 3.7, 2,8, 1.9, 0.9 and 0.4 µL L^−1^ air to *P. uviferum* EO, and 15.5, 11.6, 7.8 and 3.9 µL L^−1^ air to *B. cruckchanksii* EO into the jars. The blank jars had no EO on the tulle piece. Ten horn flies collected according to Section 4.1, were transferred into each testing flask and these were sealed with a flip-top lid. The jars were kept at 25 ± 2 °C with variable humidity (30–50%), under a light:dark cycle of 12:12 h. After 1 h and 4 h of exposure, mortality was recorded. Knock-down flies were considered dead. Mortality values were used to calculate the lethal dose for the death of 50% flies (LC_50_). Each assay was replicated three times.

#### 4.4.4. Olfactometer Bioassays

A dual-port Y-tube glass olfactometer (stem, 110 mm; ports, 90 mm at a 130° angle from the stem; internal diameter, 10 mm) was used to assess the olfactory response of *H. irritans* [31,87] to the EOs. Each port of the Y-tube was connected to a Pasteur pipette, each containing either the sample or the control. A quantity of 10 μL of sample at 1 μg of EO per μL of acetone was applied to a piece of filter paper (0.5 cm × 5 cm). Acetone (10 μL; liquid chromatography grade; LiChrosolv^®^, Merck KGaA, Darmstadt, Germany) was applied for the control. The filter paper was air-dried and placed in the middle of a Pasteur pipette connected to a port of the olfactometer. The base of the stem was connected to a vacuum pump generating a purified air flow (800 mL min^−1^) to carry the volatile stimuli from the ports to the stem. The air was purified through a column of activated carbon. Unsexed horn flies were starved for 24 h prior to testing. To test the olfactory response of the flies to samples, horn flies were released into the olfactometer individually through a hole at 2 cm of the base of the stem, and immediately afterward it was sealed with a Teflon cap and given 3 min for choosing between the sample or control. Their presence in the sample-treated or control port (>half of the port) was recorded. If the fly did not make any choice, it was discarded. Each sample was tested with 10 different flies, sequentially introduced into the olfactometer. After two flies were tested, the Y-tube was cleaned with ethanol followed by acetone, and air-dried. Additionally, the order of ports was randomized. Each test was replicated 6 times. Responses were recorded as the percentage of flies inside the treatment or control ports.

#### 4.4.5. Statistical Analyses

The statistical software Statistix 10 (Analytical Software, Tallahassee, FL, USA) was used to analyze the data. The Shapiro–Wilk test was used to test whether data conformed to a normal distribution. The differences in the antifeedant activity and olfactory response among treatments on *H. irritans* were analyzed using a two-way ANOVA test (*p* ≤ 0.05) with a post-hoc Tukey HSD test. The olfactory response among treatments on *H. irritans* were analyzed using a one-way ANOVA test (*p* ≤ 0.05, *p* ≤ 0.01) with a post-hoc Tukey HSD test. The results were expressed as means with their corresponding standard errors. The statistical software IMB SPSS Statistics for Windows, v. 28.0 (IBM Corp., Armonk, NY, USA) was used to analyze the dose–mortality response of the EOs. The mean mortality data of the three replicates per dose (5–9 doses per EO) were used to calculate the medium lethal dose (LC_50_) values by means of a Probit regression (Harvard Programming; Hg1, 2).

## Figures and Tables

**Table 1:** Chemical composition of the *B. cruckchanksii* leaves essential oil (EOs).

RT	RI	Compound	%	Identification
9.50	924	*α*-Thujene	0.37	RI, MS
9.65	930	*α*-Pinene	36.50	RI, MS, Co-I
10.87	969	*β*-Pinene	0.81	RI, MS, Co-I
12.66	1027	Limonene	20.50	RI, MS, Co-I
21.93	1350	*α*-Cubebene	1.16	RI, MS
22.49	1371	Ylangene	0.07	RI, MS
22.62	1376	Copaene	1.18	RI, MS
23.02	1390	*β*-Elemene	0.24	RI, MS
23.42	1406	*α*-Gurjunene	2.59	RI, MS
23.63	1414	Caryophyllene	4.63	RI, MS
23.96	1428	*β*-Gurjunene	0.20	RI, MS
24.17	1437	Aromadendrene	1.43	RI, MS
24.38	1445	Bicyclosesquiphellandrene	0.82	RI, MS
24.51	1450	*α*-Caryophyllene	0.86	RI, MS
24.65	1456	Alloaromadendrene	1.19	RI, MS
24.96	1468	*β*-Cadinene	1.23	RI, MS
25.31	1482	*α*-Selinene	1.48	RI, MS
25.52	1490	Ledene	2.04	RI, MS
25.71	1498	δ-Cadinene	3.63	RI, MS
25.89	1505	γ-Cadinene	0.21	RI, MS
26.06	1513	Calamenene	8.69	RI, MS
26.33	1525	Cubenene	5.65	RI, MS
26.62	1537	*α*-Calacorene	0.21	RI, MS
27.48	1574	Spathulenol	0.81	RI, MS
27.65	1581	Caryophyllene oxide	0.60	RI, MS
28.04	1597	Ledol	0.21	RI, MS
28.17	1603	Humulene-1,2-epoxide	1.10	RI, MS
28.59	1622	Epicubenol	0.40	RI, MS, Co-I
28.91	1637	Cubenol	1.18	RI, MS, Co-I
		Monoterpene hydrocarbons	58.18	
		Sesquiterpene hydrocarbons	37.52	
		Oxygenated sesquiterpenes	4.30	
		**Total sesquiterpenes**	**41.82**	

RT: Retention time (min), RI: Kovats retention index, %: Considering detected compounds, Co-I: co-injection.

**Table 2:** Toxicological activity of *B. cruckchanksii* leaves and *P. uviferum* heartwood essential oils against horn flies.

EO	Time (h)	LC_50_ (95% FCI) (µL L^−1^ Air)	χ^2^	*p*	Slope (±SE)
*B. cruckchanksii*	1	-	-	-	-
	4	3.583 (0.003–6.146)	0.329	0.028	1.167 ± 0.531
*P. uviferum*	1	9.414 (6.438–18.348)	1.672	<0.001	1.287 ± 0.241
	4	1.016 (0.553–1.494)	2.810	<0.001	1.140 ± 0.198

LC_50_: Dose causing 50% mortality; FCI—fiducial confidence interval; χ^2^: chi-square value.

## Data Availability

Not applicable.

## Sample Availability

Samples of the essential oils are not available from the authors.
